# Integrative analysis reveals the prognostic value and functions of splicing factors implicated in hepatocellular carcinoma

**DOI:** 10.1038/s41598-021-94701-8

**Published:** 2021-07-26

**Authors:** Yue Wang, Fan Yang, Jiaqi Shang, Haitao He, Qing Yang

**Affiliations:** Department of Pathogenobiology, College of Basic Medical Sciences, Jilin University, 126 Xinmin Street, Changchun, 130021 Jilin Province China

**Keywords:** Cancer, Systems biology

## Abstract

Splicing factors (SFs) play critical roles in the pathogenesis of various cancers through regulating tumor-associated alternative splicing (AS) events. However, the clinical value and biological functions of SFs in hepatocellular carcinoma (HCC) remain obscure. In this study, we identified 40 dysregulated SFs in HCC and established a prognostic model composed of four SFs (DNAJC6, ZC3H13, IGF2BP3, DDX19B). The predictive efficiency and independence of the prognostic model were confirmed to be satisfactory. Gene Set Enrichment Analysis (GSEA) illustrated the risk score calculated by our prognostic model was significantly associated with multiple cancer-related pathways and metabolic processes. Furthermore, we constructed the SFs-AS events regulatory network and extracted 108 protein-coding genes from the network for following functional explorations. Protein–protein interaction (PPI) network delineated the potential interactions among these 108 protein-coding genes. GO and KEGG pathway analyses investigated ontology gene sets and canonical pathways enriched by these 108 protein-coding genes. Overlapping the results of GSEA and KEGG, seven pathways were identified to be potential pathways regulated by our prognostic model through triggering aberrant AS events in HCC. In conclusion, the present study established an effective prognostic model based on SFs for HCC patients. Functional explorations of SFs and SFs-associated AS events provided directions to explore biological functions and mechanisms of SFs in HCC tumorigenesis.

## Introduction

Hepatocellular carcinoma (HCC) is a kind of malignancy originating from liver parenchymal cells, accounting for 75–90% of primary liver cancer. Currently, HCC remains the major cause of morbidity and mortality among malignant cancers worldwide^1,2^. Hepatitis B/C virus infection, smoking, drinking, exposure to aflatoxin and thorium dioxide are known risk factors of HCC^3,4^. Due to the asymptomatic nature of HCC, most individuals were diagnosed at advanced stages, with high rates of metastasis, recurrence, and mortality, even more with limited treatment options^5^. In addition, conventional clinicopathological characteristics cannot precisely predict the prognoses of HCC patients due to the heterogeneity and pathogenic complexity of HCC^6^. It is valuable to clarify molecular mechanisms underlying the pathogenesis of HCC and develop novel molecules to be diagnostic, therapeutic, and prognostic targets for HCC patients.

Alternative splicing (AS) is a vital post-transcriptional regulation mechanism in eukaryotes. The process of splicing is mediated by core spliceosome and hundreds of splicing-associated proteins, which were classified as splicing factors (SFs)^7^. Generally, SFs orchestrate various RNA splicing via recognizing cis-regulatory elements within the alternative exons or flanking introns^8^. The expression alternations or mutations of SFs can result in aberrant landscape of AS events and further affect downstream protein production^9^. As important regulators of AS events, SFs play essential roles in the occurrence and progression of HCC. Generally, mutations of SF genes occur mutually exclusive of each other^10^. However, the global expression patterns of SFs in HCC remain unclear. Previous studies mainly focused on the biological functions and clinical value of specific SFs. For example, MBNL3, an oncofetal splicing factor, is upregulated in HCC tissues and induces exon 4 inclusion of lncRNA-PXN-AS1. The transcript of lncRNA-PXN-AS1 containing exon 4 can increase PXN mRNA expression through binding to the 3’ UTR of PXN mRNA and protecting it from miR-24 induced degradation, thereby promoting tumorigenesis of HCC and indicating poor prognosis of HCC patients^11^. SF3B1, the central spliceosome component, is overexpressed in HCC. The overexpression of SF3B1 alters the splicing pattern of KLF6 and is closely correlated with poor prognosis of HCC patients^12^. It is of great significance to explore the overall expression abnormalities, prognostic value and corresponding biological functions of SFs in HCC.

In this study, we systemically analyzed the expression alterations of SFs and their prognostic values in HCC using gene expression profile downloaded from liver hepatocellular carcinoma (LIHC) of the Cancer Genome Atlas (TCGA). A prognostic model based on SFs for HCC patients was constructed, and its prognostic capacity was validated to be good. Gene set enrichment analysis (GSEA) was conducted to investigate underlying mechanisms associated with the prognostic model. In addition, we identified aberrant spliced AS events and prognostic AS events in HCC. The correlations between the 4 SFs in the model and prognostic AS events were analyzed to construct SFs-AS events regulatory network. Then functional analysis of protein-coding genes of AS events involved in the SFs-AS regulatory network further indicated the potential biological functions of AS events regulated by SFs in the prognostic model. Taken together, our present study provided a novel prognostic indicator for HCC patients and explored the potential functions of SFs implicated in HCC through regulating AS events.

## Materials and methods

### Data collection and processing

Gene expression counts data and clinical information of LIHC were downloaded from TCGA data portal (http://tcgadata.nci.nih.gov/tcga/)^13^. The gene counts data were converted and subsequently standardized using R package “DESeq2”^14^, from which mRNA expression profile were obtained and annotated according to gene annotation file (GTF) of human downloaded from “http://ftp.ensembl.org/pub/release-103/gtf/homo_sapiens/”. A total of 404 SF genes were identified through aggregating with the following gene sets: (1) SF-related gene sets (KEGG_SPLICEOSOME, REACTOME_MRNA_SPLICING, and REACTOME_MRNA_SPLICING_MINOR_ PATHWAY) downloaded from version 7.0 of Molecular Signature Database (MSigDB, org/gsea/msigdb/index.jsp); (2) SF-related genes downloaded from SpliceAid 2 (http://193.206.120.249/splicing_tissue.html)^15^. The list of 404 SFs were provided in Supplementary Table S1. Then the expression profile of SFs was extracted from mRNA expression profile of HCC.

We downloaded the percent spliced in (PSI) value of splicing events of HCC from TCGA SpliceSeq (https://bioinformatics.mdanderson.org/TCGASpliceSeq), a data portal providing systematic profiles of AS events for all TCGA disease types^16^. PSI value represents the ratio of inclusion/exclusion normalized read counts to the total (both inclusion and exclusion) normalized read counts for a particular splicing pattern. Each AS event was assigned a unique annotation consisting of gene symbol, ID number, and splicing type. To ensure the credibility and universality of the present study, the AS events were filtered according to the following criteria: (1) the AS events with more than 75% effective PSI value; (2) the average of PSI value ≥ 0.05.

### Identification of differentially expressed SFs and aberrant spliced AS events in HCC

The expression of SFs between 50 paired tumor tissues and normal adjacent tissues of HCC were compared using R package of “limma”^17^. SFs with absolute value of log2-fold change (|log2FC|) ≥ 0.5 and adjusted *P*-values < 0.05 were considered significantly differentially expressed, in which *P*-values were adjusted using the Benjamini–Hochberg (BH) correction. The PSI value distributions of AS events between 50 normal adjacent tissues and 371 tumor tissues of HCC were compared by Wilcoxon rank-sum test to evaluate the splicing pattern alterations in HCC tissues. AS events with *P* value < 0.05 were considered differentially spliced. UpSet plot and Venn plot, generated by R package of “UpSetR” and “yyplot” respectively, were used to qualitatively visualize the intersecting gene sets among the types of differentially spliced AS events.

### Screening for prognostic SFs and AS events in HCC

A total of 342 HCC patients with follow-up time ≥ 30 days were included to perform univariate Cox regression analysis for dysregulated SFs and differentially spliced AS events. SFs with *P* < 0.05 were confirmed as prognosis-associated SFs. The hazard ratios (HRs) and 95% confidence interval (95% CI) of prognosis-associated SFs in HCC were visualized using R package of “forestplot”. AS events with *P* < 0.05 were considered significantly correlated with the overall survival (OS) of HCC. Then UpSet plot and Venn plot, generated using R package of “UpSetR” and “yyplot” respectively, were applied to qualitatively display the intersecting gene sets among the types of prognostic AS events.

### Construction of the prognostic risk score model based on SFs for HCC patients

Among the prognosis-associated SFs, the least absolute shrinkage and selection operator (LASSO) regression was conducted by R package “glmnet” to remove highly correlated SFs and prevent overfitting. Then 171 HCC patients were randomly selected to be training set, remaining 171 patients as validating set. The demographic information and clinical characteristics between training set and validating set were compared through χ^2^ test or Fisher’s exact test to ensure the random distribution between training set and validating set, with *P* < 0.05 considered statistically significant. Multivariate Cox analysis was applied using R package of “survival” to construct an optimal prognostic risk score model based on expression of SFs in training set, in which the risk scores of HCC patients were computed by the following formula: risk score = (β_SF1_ × expression level of SF1) + (β_SF2_ × expression level of SF2) + ⋯ + (β_SFn_ × expression level of SFn). The median of risk scores in training set was set as cut-off value to stratified patients as low-risk and high-risk subgroup.

### Identification the efficiency and independence of the prognostic model

We performed the log-rank test and Kaplan–Meier survival analysis using “survival” and “survminer” packages in R to explore the statistical difference of OS between HCC patients in low-risk and high-risk subgroups. The sensitivity and specificity of the prognostic model was evaluated by receiver-operating characteristic (ROC) analysis. Then univariate and multivariate Cox regression analyses were performed to investigate the independent predictive value of the prognostic model compared with demographic information and clinical characteristics including age, gender, American Joint Committee on Cancer (AJCC) stage, tumor size, lymph node, metastasis status, and vital status. R package of “forestplot” was used to visualize the results of univariate and multivariate Cox regression analyses.

### GSEA

To explore the potential pathways and gene sets associated with the constructed prognostic model, GSEA was performed using R package “GSEABase” to find enriched terms in the canonical pathways (C2) collected from the Kyoto Encyclopedia of Genes and Genomes (KEGG); in the ontology gene sets (C5) derived from the gene ontology resource (GO) consisting of biological process (BP), cellular component (CC), and molecular function (MF); and the oncogenic signatures gene sets (C6) which were often dysregulated in cancer. All gene sets above (C2, C5, and C6) were retrieved from Molecular Signature Database (MsigDB v6.2). It was considered significantly enriched when *P* < 0.01 and false discovery rate (FDR) q < 0.05. The results of GSEA were visualized by R package of “clusterProfiler”.

### Construction of prognostic SFs-AS events regulatory network

Spearman correlation analysis was performed to analyze the correlation of expression of SFs in the prognostic model and PSI value of prognostic AS events. It was considered that SFs and AS events were significantly correlated when correlation coefficient r > 0.4 (or <  − 0.4) and *P* < 0.01. Then the potential regulatory network of SFs and AS events was visualized by Cytoscape (version 3.7.2).

### Protein–protein interaction (PPI) network analysis and functional enrichment analysis

According to the human gene annotation file downloaded from http://asia.ensembl.org/index.html, protein-coding genes were screened out from genes of the AS events involved in the prognostic SFs-AS events regulatory network. To explore potential interactions among these protein-coding genes, we uploaded these protein-coding genes to the STRING database (https://string-db.org/), a biological database presenting functional protein association networks. Then the PPI network was set up with the identified genes by integrating the data retrieved from the STRING database. Results of PPI network analysis were visualized and analyzed via Cytoscape (version 3.7.2). Top 10 hub genes were identified through calculating the nodes’ scores by cytoHubba. R package of “ClusterProfiler” was used to perform the GO enrichment analysis and KEGG pathway analysis for these protein-coding genes^18–20^.

## Results

### Identification the differentially expressed SFs in HCC

**Figure 1:**
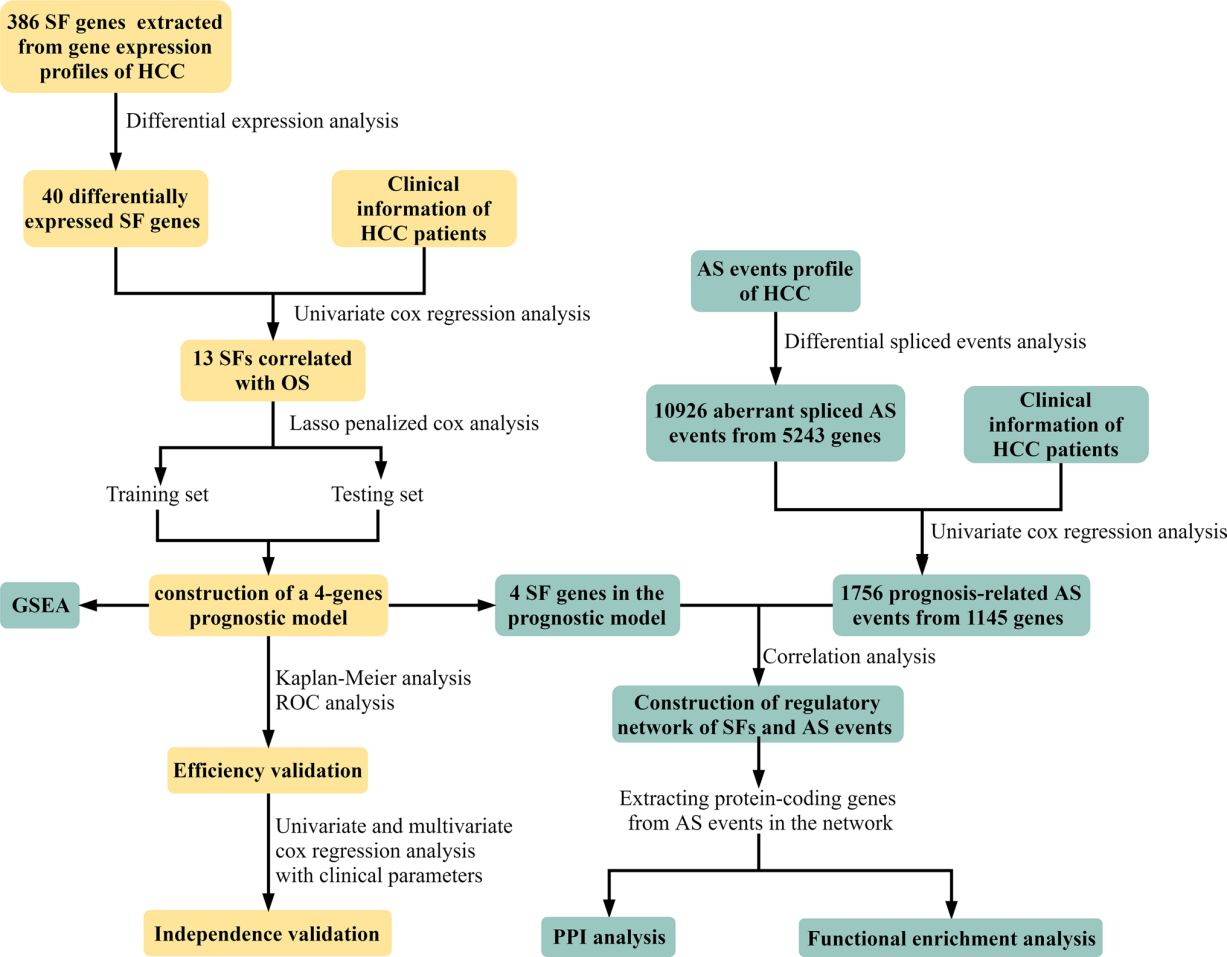
The approach and workflow for the exploration of clinical value and molecular functions of splicing factors (SFs) in HCC. The flowchart was drawing using visio2013 (https://products.office.com/en/visio/flowchart-software).

**Figure 2:**
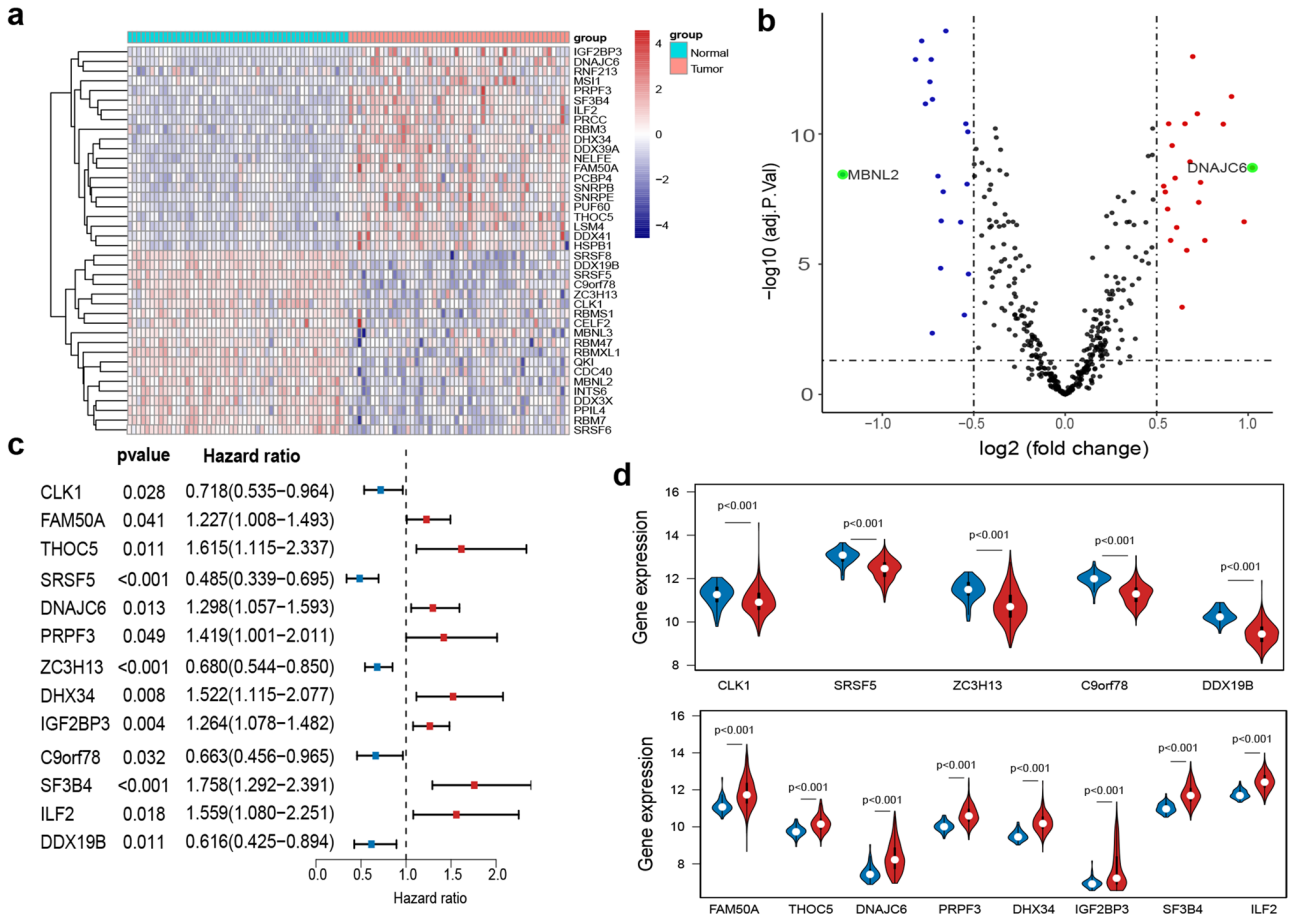
Identification of dysregulated SFs and survival-associated SFs in HCC. (**a**) Heatmap of differentially expressed SFs between 50 pairs of normal tissues and tumor tissues of HCC (|log2FC|≥ 0.5, adjusted *P* < 0.05). (**b**) Volcano plot of differentially expressed SFs in HCC. The red and blue dots represent upregulated and downregulated SFs respectively; the green dots represent the dysregulated SFs with |log2FC|≥ 1; the black dots represent the SFs with no significant difference. (**c**) Forest plot of hazard ratios for survival-associated SFs in HCC. The red and blue boxes represent risk factors or protective factors of HCC, respectively. (**d**) Violin plots showing the expression of survival-associated SFs in 50 normal tissues (blue) and 371 HCC tissues (red). SFs presented in upper were protective factors for HCC patients; SFs presented in lower were risk factors for HCC patients.

The approach and workflow of this study was illustrated in Fig. 1. To investigate the expression alterations of SFs in HCC, we compared the expression of 404 SF genes between 50 paired normal tissues and HCC tissues and identified 40 differentially expressed SFs in HCC tissues, among which 21 were upregulated and 19 were downregulated (Table 1). Hierarchical clustering analysis confirmed the significant differences in expression patterns of differentially expressed SFs between normal and tumor tissues of HCC (Fig. 2a). In addition, volcano plot displayed the distribution of differentially expressed SFs (Fig. 2b).

**Table 1:** The dysregulated SFs in HCC tumor tissues compared with adjacent normal tissues.

Gene symbol	t	*P* value	Adjusted *P* value	log2FoldChange
MBNL2	− 7.7405	2.80E−10	3.61E−09	− 1.216390192
SRSF8	− 11.0985	1.79E−15	1.38E−13	− 0.818440611
SRSF5	− 11.8659	1.39E−16	2.68E−14	− 0.784565905
DDX19B	− 9.79444	1.62E−13	6.97E−12	− 0.764105134
C9orf78	− 10.4671	1.55E−14	9.96E−13	− 0.739616046
QKI	− 11.1391	1.56E−15	1.38E−13	− 0.732476871
MBNL3	− 3.25834	0.001953	0.0044613	− 0.726697915
DDX3X	− 9.93991	9.73E−14	4.69E−12	− 0.725762803
RBMXL1	− 7.67623	3.56E−10	4.16E−09	− 0.695428933
RBMS1	− 5.18429	3.42E−06	1.43E−05	− 0.680954214
ZC3H13	− 6.47666	3.06E−08	2.19E−07	− 0.677839203
INTS6	− 7.25235	1.72E−09	1.66E−08	− 0.666948085
RBM7	− 12.3505	2.88E−17	1.11E−14	− 0.652489521
CLK1	− 6.41436	3.85E−08	2.44E−07	− 0.571390031
CELF2	− 3.84517	0.000323	0.0008978	− 0.551245241
SRSF6	− 9.22304	1.24E−12	4.07E−11	− 0.543142739
PPIL4	− 8.92578	3.64E−12	8.26E−11	− 0.532270648
RBM47	− 5.02466	6.02E−06	2.40E−05	− 0.528956611
PRPF3	7.39948	9.93E−10	1.01E−08	0.538658868
DDX41	7.240208	1.79E−09	1.69E−08	0.547251578
PUF60	6.785813	9.71E−09	7.65E−08	0.560149209
THOC5	9.196583	1.37E−12	4.07E−11	0.565605055
RBM3	5.927696	2.32E−07	1.22E−06	0.5764643
PRCC	8.560312	1.37E−11	2.79E−10	0.584721824
SNRPB	7.621772	4.35E−10	4.94E−09	0.600985254
LSM4	6.249725	7.08E−08	3.90E−07	0.609645727
MSI1	4.092934	0.000145	0.00044808	0.639140638
ILF2	9.196966	1.37E−12	4.07E−11	0.654889388
RNF213	5.662538	6.10E−07	2.94E−06	0.664719245
SNRPE	8.105903	7.28E−11	1.17E−09	0.682291603
DHX34	11.32695	8.30E−16	1.07E−13	0.697395517
SF3B4	9.516833	4.35E−13	1.68E−11	0.722488238
FAM50A	6.956319	5.15E−09	4.23E−08	0.730040853
PCBP4	7.510873	6.57E−10	7.24E−09	0.739950592
IGF2BP3	5.931286	2.29E−07	1.22E−06	0.763620084
NELFE	9.167253	1.52E−12	4.20E−11	0.864302671
DDX39A	10.049	6.63E−14	3.66E−12	0.909431813
HSPB1	6.443956	3.45E−08	2.36E−07	0.978176356
DNAJC6	7.931304	1.39E−10	1.98E−09	1.02334043
CDC40	− 7.46249	7.86E−10	8.43E−09	9.134968345

*SFs* splicing factors, *HCC* hepatocellular carcinoma.

### Construction of a prognostic model based on SFs for HCC patients

**Figure 3:**
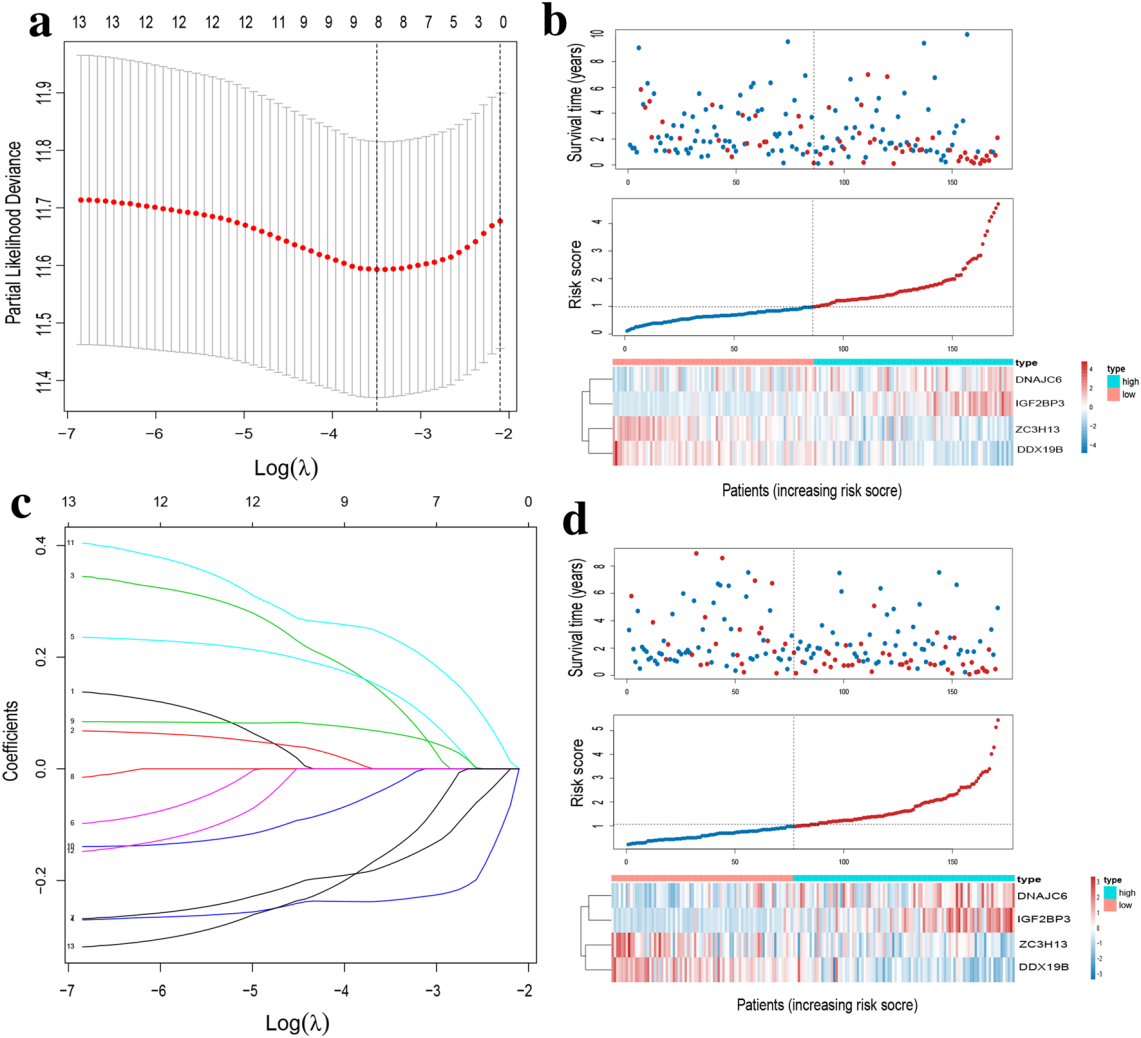
Construction of the prognostic risk score model based on SFs for HCC patients. (**a**) Selection of tuning parameter (λ) in the LASSO regression analysis via tenfold cross-validation. The dotted vertical lines were plotted at the optimal λ values based on the minimum criteria and 1 standard error of the minimum criteria. (**b**) LASSO coefficient profiles of the 13 survival-associated SFs. The dotted vertical line was plotted at the same position as the dotted vertical line in (**a**), producing 8 nonzero coefficients (THOC5, SRSF5, DNAJC6, ZC3H13, IGF2BP3, C9orf78, SF3B4, and DDX19B). (**c**) Risk plot of HCC patients in the training set; (**d**) risk plot of HCC patients in the validating set. For (**d,e**), Upper part assembly indicated the distribution of HCC patients’ survival status and survival times ranked by risk score; the middle part represented the increasing risk score curve, in which HCC patients were divided into low-risk (blue) and high-risk (red) subgroup according to the median value of risk scores of patients in the training set; and the bottom heatmap displayed expression pattern of SFs involved in the prognostic model.

The relationship between the 40 dysregulated SFs and the prognosis of 342 HCC patients with follow-up time ≥ 30 days were analyzed by univariate Cox analysis, identifying 13 significantly prognosis-associated SFs (Fig. 2c). Among the 13 prognosis-associated SFs, 5 SFs with hazard ratio (HR) < 1 (CLK1, SRSF5, ZC3H13, C9orf78, DDX19B) were considered protective factors; while the remaining 8 SFs with HR > 1 (FAM50A, THOC5, DNAJC6, PRPF3, DHX34, IGF2BP3. SF3B4, IL2) were considered risk factors. As expected, SFs as protective factors of HCC were significantly downregulated in HCC tissues (Fig. 2d, upper); while SFs as risk factors of HCC were upregulated in HCC tissues (Fig. 2d, lower), indicating their clinical potential as diagnostic, therapeutic, and prognostic biomarkers for HCC patients. Therefore, we applied LASSO regression analysis to the 13 prognostic SFs and identified 8 more valuable prognostic SFs (THOC5, SRSF5, DNAJC6, ZC3H13, IGF2BP3, C9orf78, SF3B4, and DDX19B) (Fig. 3a,b).

Following, to easily and reliably stratify outcomes of HCC patients with SFs, we randomly categorized 342 HCC patients into training set and validating set. Except the gender, no clinical parameter was significantly different between training set and validating set, identifying their random distribution (Table 2). In training test, the stepwise multivariate Cox regression was applied and a total of 4 SFs (DNAJC6, ZC3H13, IGF2BP3, and DDX19B) were selected to construct the final prognostic risk score model The normalized expression of these 4 SFs and their corresponding coefficients, displayed in Table 3, were used to calculate risk scores for HCC patients with the following risk score calculation formula: risk score = (0.28336 × DNAJC6 expression) + (− 0.4438 × ZC3H13 expression) + (0.226331 × IGF2BP3 expression) + (− 0.63347 × DDX19B expression). Then HCC patients were divided into high-risk and low-risk subgroup based on the median value (0.9856) of the risk scores of HCC patients in training set. The distribution of survival status, risk scores, and expression patterns of SFs (DNAJC6, ZC3H13, IGF2BP3, and DDX19B) in training set and validating set were respectively visualized in Fig. 3c,d. Taken together, we constructed a 4-gene prognostic signature through univariate Cox analysis, LASSO regression analysis, and multivariate Cox analysis for differentially expressed SFs in HCC tissues.

**Table 2:** Clinical characteristics of HCC patients in TCGA.

Characteristics	Training set (n = 171)	Validating set (n = 171)	*P*-value
**Age**	59.9 ± 12.4	58.8 ± 14.0	0.431
**Gender**			0.008
Female	43	66	
Male	128	105	
**AJCC stage**			0.998
Stage I	79	82	
Stage II	39	38	
Stage III	40	39	
Stage IV	2	2	
NA	11	11	
**Tumor size**			0.989
T1	83	85	
T2	42	42	
T3	38	36	
T4	7	6	
TX	1	2	
**Lymph node**			0.102
N0	115	123	
N1	0	3	
NX	56	45	
**Metastasis status**			0.499
M0	126	118	
M1	2	1	
MX	43	52	
**Vital status**			0.498
Live	113	107	
Dead	58	64	

*HCC* hepatocellular carcinoma, *TCGA* The Cancer genome atlas, *AJCC* American Joint Committee on Cancer.

**Table 3:** The final prognostic risk score model for HCC patients.

SF_ID	HR	95% CI	P value	Coefficient
DNAJC6	1.327583	0.928473–1.898253	0.120381	0.28336
ZC3H13	0.641591	0.459594–0.895658	0.009124	− 0.4438
IGF2BP3	1.253991	0.9535–1.649179	0.105384	0.226331
DDX19B	0.530746	0.287634–0.979341	0.042687	− 0.63347

*HCC* hepatocellular carcinoma, *SF* splicing factor, *HR* hazard ratio, *CI* confidence interval.

### Identification the efficiency and independence of the prognostic model for HCC patients

**Figure 4:**
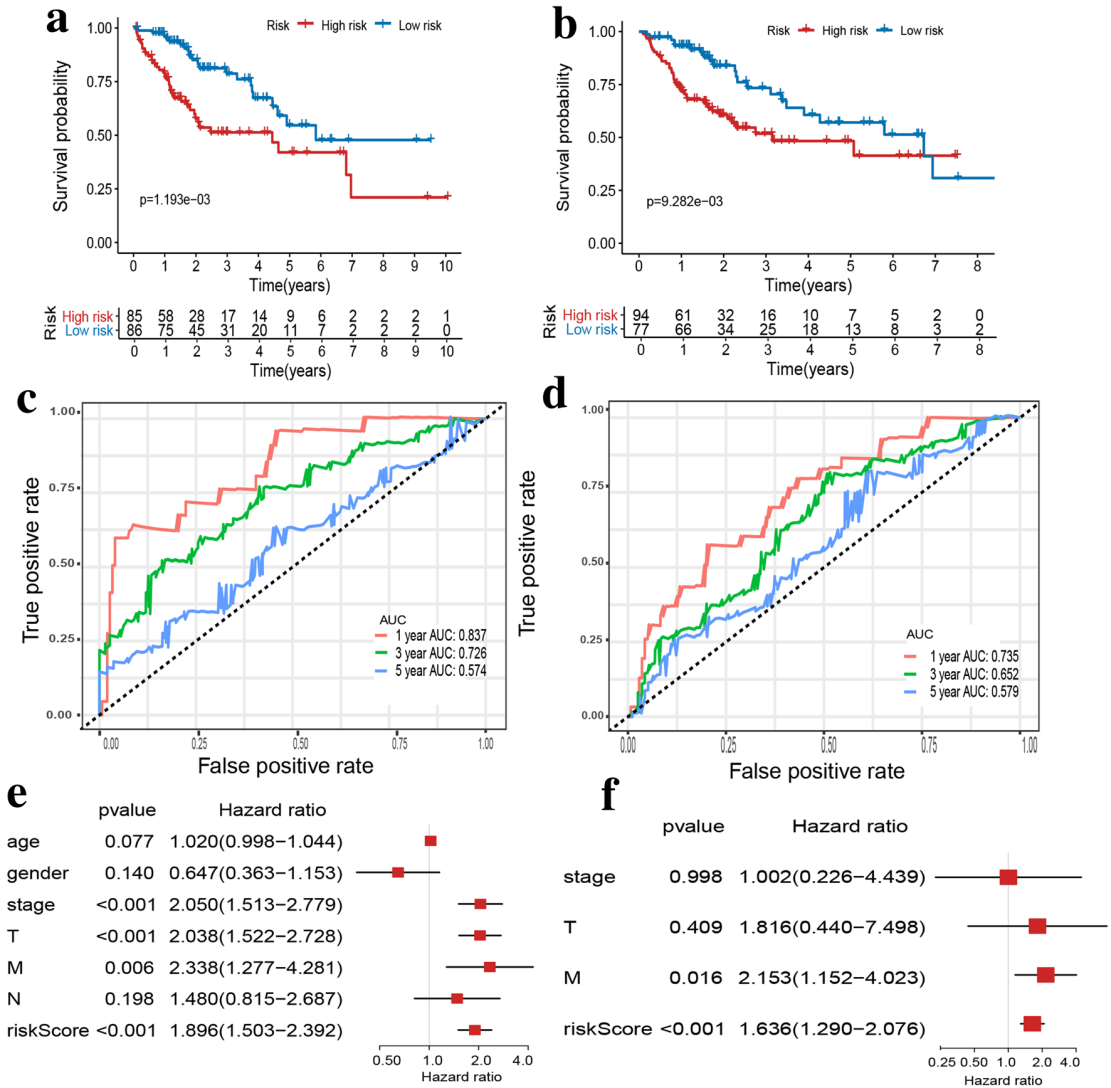
Identification the efficiency and independence of the prognostic risk score model based on SFs. (**a,b**) Kaplan–Meier analysis of the prognostic risk score model for HCC patients in training set and validating set, respectively. (**c,d**) ROC curve for HCC patients in training set and validating set respectively. (**e,f**) Univariate and multivariate analyses of the risk level calculated by the prognostic model, clinical factors and pathological characteristics with OS of HCC patients.

To probe the relationship between the risk score computed by our prognostic model and OS of HCC patients, Kaplan–Meier analysis was performed and confirmed the OS of HCC patients in high-risk group was much shorter than those in low-risk group in both training set and validating set (Fig. 4a,b). In the training set, the area under the curve (AUC) value of ROC curve for 1, 3, 5-year-survival were 0.837, 0.726, and 0.574, respectively. In the validating set, the AUC value for 1, 3, 5-year-survival of ROC curve were 0.735, 0.652, and 0.579, respectively (Fig. 4c,d). These results confirmed the high efficiency of the prognostic model in predicting 1, 3-year survival for HCC patients. To further validate the independent predictive power of the model for HCC patients, the univariate Cox regression analysis was applied and identified that risk score calculated our prognostic model, AJCC stage, tumor size, and metastasis status were risk factors of HCC patients (Fig. 4e). Then, these risk factors were incorporated into multivariate Cox hazard regression analysis, validating risk score and metastasis status as independent prognostic factors for HCC (Fig. 4f). Collectively, these results demonstrated the prognostic signature owned good prognostic performance for HCC.

### Functional exploration for the prognostic model based on SFs

**Figure 5:**
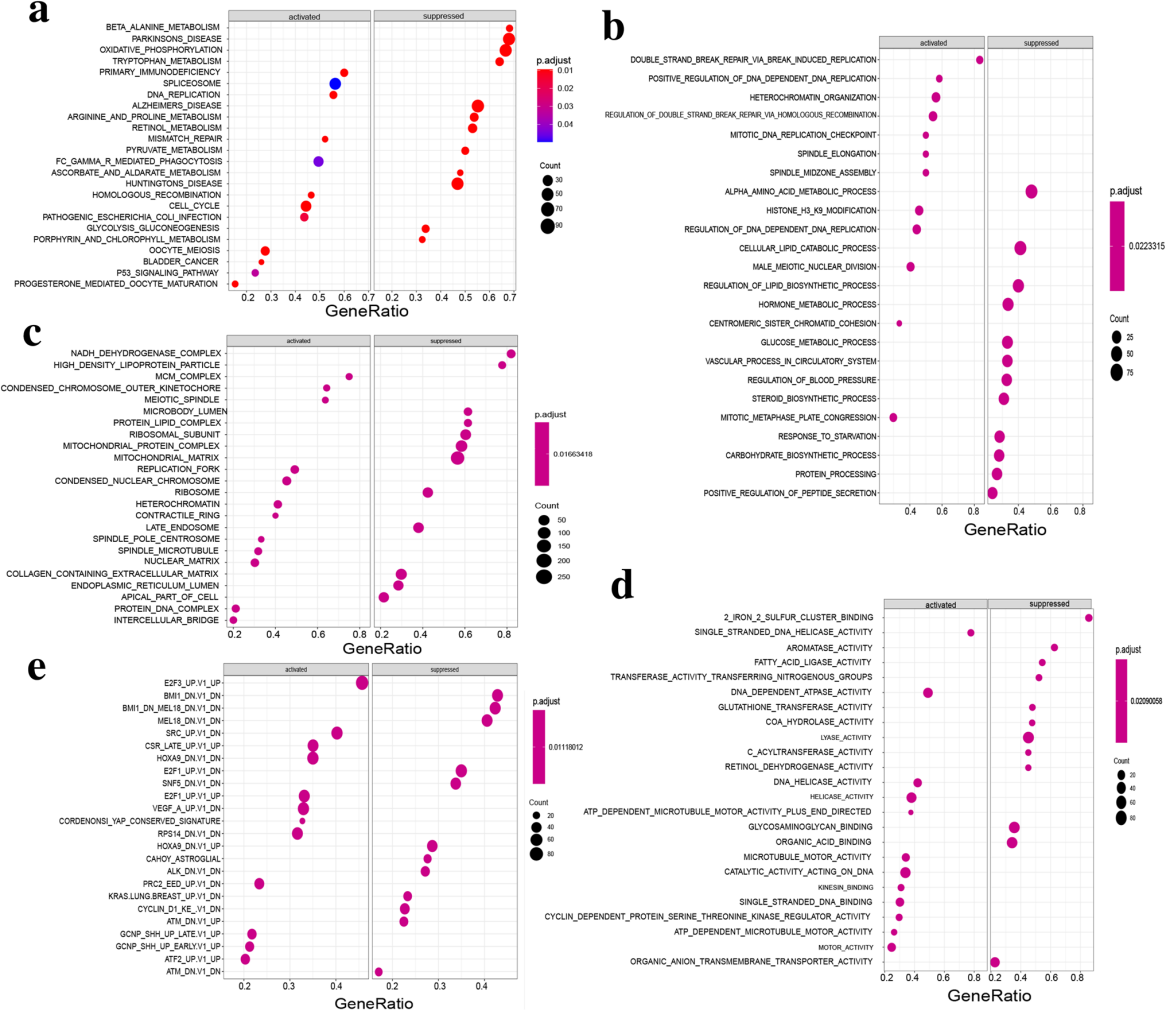
GSEA results between low-risk and high-risk group of HCC patients of the prognostic model. (**a**) Top 12 KEGG pathways activated (left) and suppressed (right) by high-risk group. (**b–d**) Top 12 gene sets of GO term activated (left) and suppressed (right) by high-risk group. Among them, results of biological process (BP) were presented in (**b**); results of cellular compartment (CC) were presented in (**c**); results of molecular function (MF) were presented in (**d**). (**e**) Top 12 gene sets of oncogenic signatures activated (left) and suppressed (right) by high-risk group. For (**a–e**), the size and color of nodes represent the number of enriched genes and adjusted *P* values.

To investigate the underlying biological functions of the prognostic model based on SFs, we utilized the mRNA expression profile and conducted GSEA between low-risk and high-risk group of HCC patients. The full results of GSEA were presented in Supplementary Table S2. In enriched KEGG pathway (C2), a great majority of cancer-related pathways were activated in high-risk group, including DNA replication, cell cycle, bladder cancer, and p53 signaling pathway, etc.; while numerous metabolism-associated pathways were suppressed in high-risk group, including β-alanine metabolism, tryptophan metabolism, retinol metabolism, and pyruvate metabolism, etc. (Fig. 5a). In enriched BP, CC, and MF of GO term (C5), top 12 gene sets activated and suppressed by high-risk group were respectively displayed in Fig. 5b–d. In enriched oncogenic signatures (C6), upregulation of multiple oncogenic genes (E2F3, E2F1, VEGFA, etc.) were activated in high-risk group; whereas downregulation of several oncogenic genes (BMI1, MEL18, and CyclinD1) were suppressed in high-risk group (Fig. 5e). Collectively, these results confirmed that high-risk score calculated by our prognostic model might confer the intense oncogenic phenotype under activation of various oncogenic genes and pathways.

### Construction of prognostic SFs-AS events regulatory network in HCC

**Figure 6:**
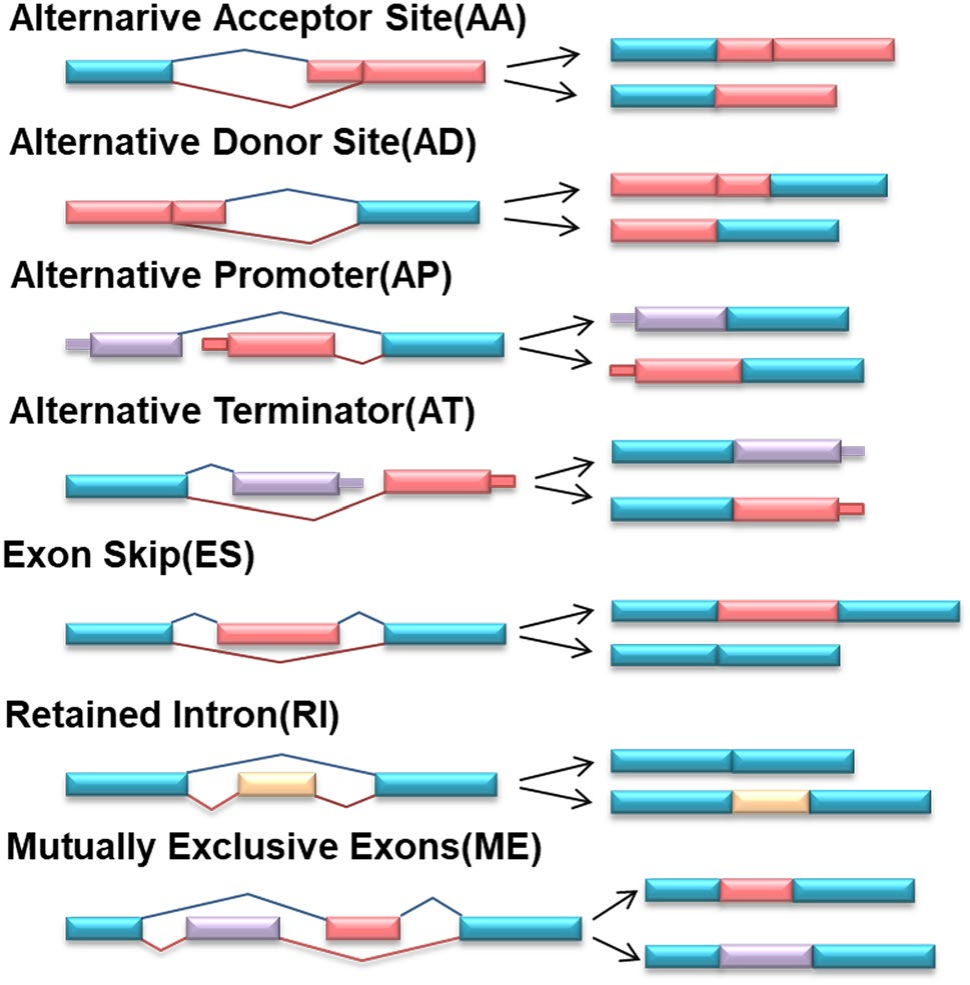
Illustration of seven types of AS events: alternative acceptor (AA), alternative donor site (AD), alternative promoter (AP), alternative terminator (AT), exon skip (ES), retained intron (RI), and mutually exclusive exons (ME).

**Figure 7:**
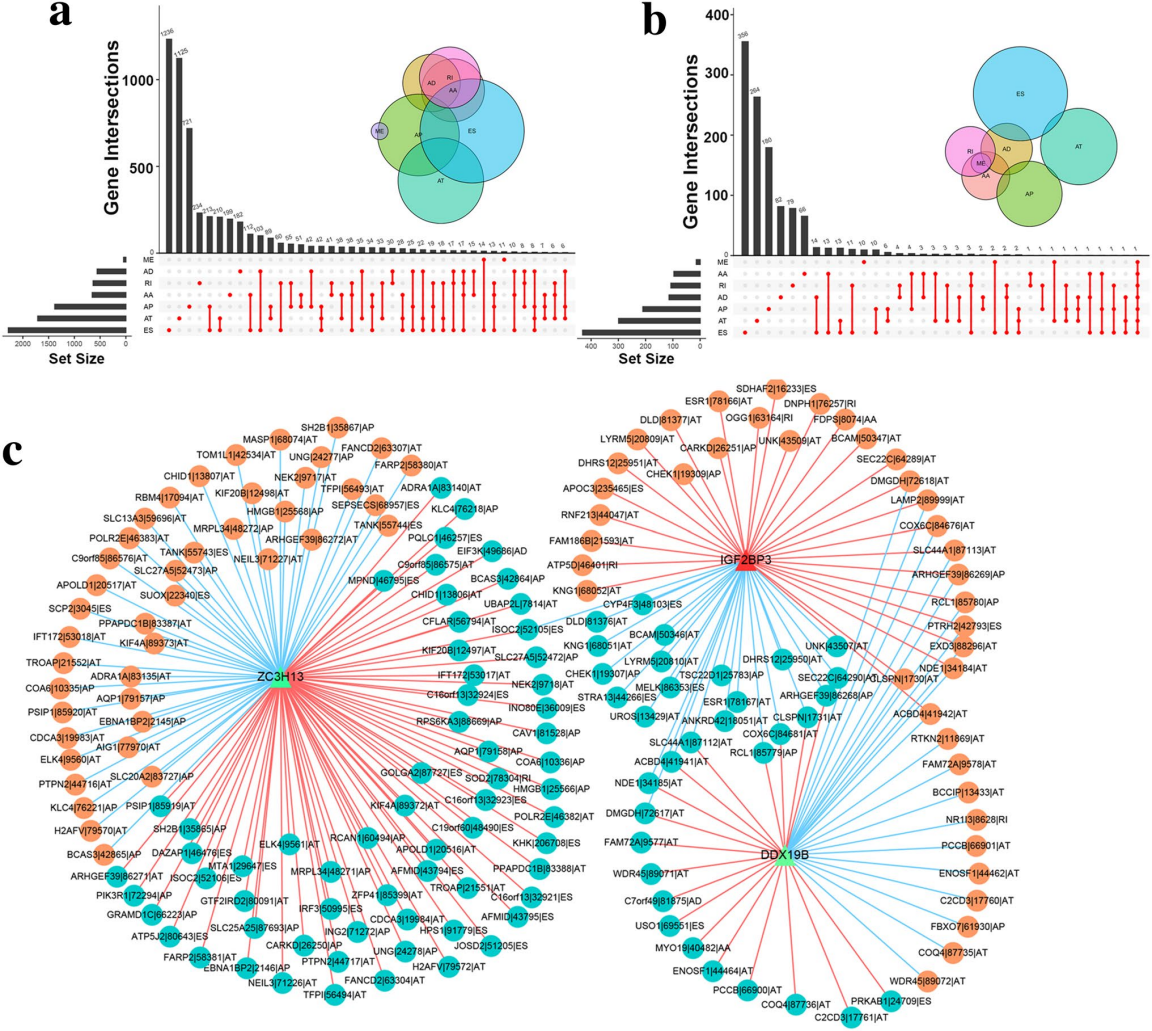
Construction of prognostic SFs-AS events regulatory network. (**a**) Upset plot and Venn diagram of parent gene interactions between the seven types of aberrant AS events in HCC. (**b**) Upset plot and Venn diagram of parent gene interactions between the seven types of prognostic AS events in HCC. (**c**) Regulatory network of SFs (ZC3H13, IGF2BP3, DDX19B) and prognostic AS events in HCC. Green triangles represent SFs that were protective factors for HCC; red triangle represents SF that was risk factor for HCC; turquoise squares represent AS events that were protective factors for HCC; orange squares represent AS events that were risk factor for HCC. The red lines represent positive correlations while the blue lines represent negative correlations.

SFs exert pro-oncogenic or antitumor effects through inducing aberrant splicing process mainly. It is meaningful to investigate regulatory relationships between SFs and AS events implicated in HCC. According to distinct splicing modes, AS events could be classified into the following seven types: alternative acceptor (AA), alternative donor (AD), alternative promoter (AP), alternative terminator (AT), exon skip (ES), retained intron (RI), and mutually exclusive exons (ME), as presented in Fig. 6. The PSI values of AS events were compared between 50 normal tissues and 371 tumor tissues of HCC. In total, 10,926 AS events from 5243 genes were identified to be altered in HCC tissues (Supplementary Table S3). The interactive gene sets among these seven types of dysregulated AS in HCC were quantitatively showed in Fig. 7a.

Then 1757 AS events from 1144 genes were confirmed to be closely associated with the prognosis of HCC patients (Supplementary Table S4). The interactive gene sets among these seven types of prognostic AS in HCC were visualized in Fig. 7b. Following we explored the correlations of expression of SFs in our prognostic model (DNAJC6, ZC3H13, IGF2BP3, DDX19B) and PSI values of prognostic AS events through Spearman correlation analysis, and identified 39 ZC3H13-associated AS events, 53 IGF2BP3-associated AS events, and 106 ZC3H13-associated AS events (Supplementary Table S5). However, no DNAJC6-associated AS events was screened out. According to the results of correlation analysis, we established the potential regulatory network of SFs and AS events in HCC (Fig. 7c). From the regulatory network, we concluded the specific transformations of AS events induced by dysregulation of ZC3H13, IGF2BP3, and DDX19B in HCC (Supplementary Table S6).

### Functional exploration for the protein-coding genes of AS events in the SFs-AS events regulatory network

**Figure 8:**
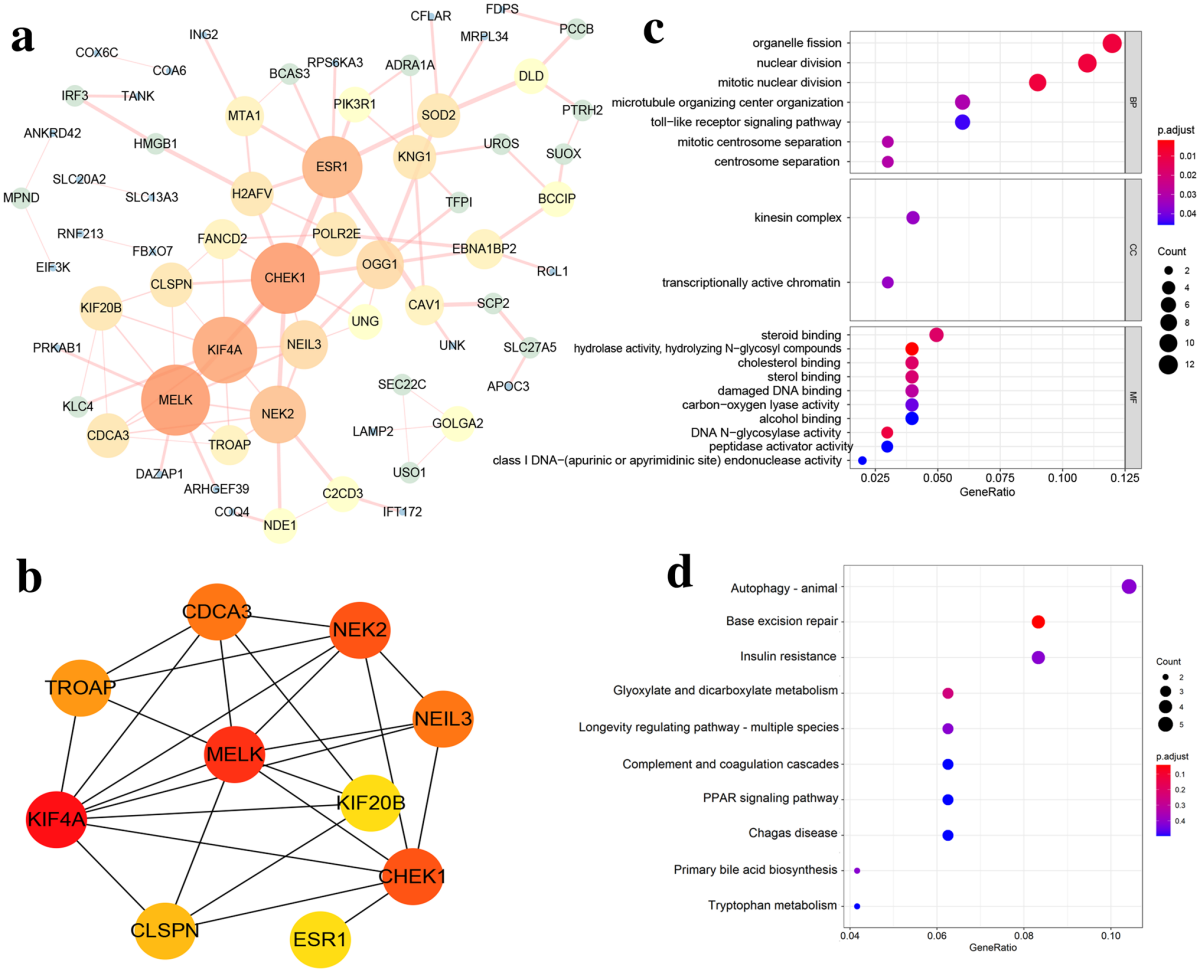
Functional exploration of AS events regulated by SFs in the prognostic model. (**a**) PPI network of 108 protein-coding genes of AS events involved in the SFs-AS events regulatory network. (**b**) The PPI network of the top 10 hub genes. (**c**) GO categories (BP, CC, and MF) enriched by above 108 protein-coding genes. (**d**) Top 10 KEGG pathways enriched by above 108 protein-coding genes. For (**c,d**), the size and color of nodes represent the number of enriched genes and adjusted *P* values.

In total, there were 180 AS events from 117 genes involved in the SFs-AS events regulatory network. Among these 117 genes, 108 genes were annotated to be protein-coding genes according to the human gene annotation file, which were listed in Supplementary Table S7. To better understand interactions among these 108 protein-coding genes, we established the PPI network by integrating the data retrieved from the STRING database (Fig. 8a). Hub genes ranking top 10 in the PPI network were selected by sorting node degree using cytoHubba in Cytoscape (Fig. 8b). These hub genes, including MELK, KIF4A, CHEK1, NEK2, NEIL3, CDCA3, TROAP, CLSPN, ESR1, and KIF20B, highly interconnected with other proteins in PPI network. Then we explored the potential biological functions of these 108 protein-coding genes by GO enrichment analysis and KEGG pathway analysis. The results of GO terms enriched by these protein-coding genes were presented in Fig. 8c and Supplementary Table S8. In BP, top three enriched terms were organelle fission, nuclear division, and mitotic nuclear division, which were essential for sustaining proliferation of cancer cells. In CC, only kinesin complex and transcriptionally active chromatin were significantly enriched. In MF, top three terms were steroid binding, hydrolase activity, hydrolyzing N-glycosyl compounds, and cholesterol binding. Besides, the results of KEGG pathways enriched by these 108 protein-coding genes were listed in Supplementary Table S8. Especially, top 10 enriched KEGG pathways were displayed in Fig. 8d, among which autophagy, PPAR signaling pathway, AMPK signaling pathway were closely related to tumor progression. Overlapping the C5 of GSEA in Supplementary Table S1 and KEGG pathways in Supplementary Table S5, seven mutual pathways were identified including glyoxylate and dicarboxylate metabolism, primary bile acid biosynthesis, complement and coagulation cascades, PPAR signaling pathway, tryptophan metabolism, propanoate metabolism, and prion disease. Therefore, we speculated ZC3H13, IGF2BP3, and DDX19B could trigger aberrant AS events and thus induce dysregulation of these 7 pathways, which might contribute to HCC progression.

## Discussion

HCC is a heterogeneous tumor originating from liver parenchymal cells. Over the last few decades, increasing database-based bioinformatics analyses have made great efforts to investigate various molecular alterations, including mRNA, lncRNAs, circular RNAs, and miRNAs, to explore their biological functions and potential key molecular mechanisms involving in the pathogenesis of HCC and screen out targets as index of diagnosis, prognosis, and therapy for HCC patients^21–24^. Recently, the significance of splicing attracted increasing attention due to its capacity of expanding genomic coding capacity and increasing protein diversity at post-transcriptional level^25^. It is worth mentioning that the choices of AS events are mainly orchestrated by SFs^26^. Increasing evidence have showed expression alterations of SFs can induce the alterations of AS events, thus triggering various oncogenic process^27,28^. It has been confirmed several dysregulated SFs were closely correlated with the prognosis of HCC patients^29–31^. However, existing studies were limited to explore the role or molecular mechanism of a single SF gene in tumor progression. It is valuable to systematically analyze the prognostic ability of SFs and establish a novel prognostic model based on SFs for HCC patients.

In present study, we established a prognostic model consisting of four SFs (DNAJC6, ZC3H13, IGF2BP3, and DDX19B), which could classify HCC patients as high-risk and low-risk subgroups. Encouragingly, Kaplan–Meier analysis of training set and validating set revealed HCC patients in low-risk group exhibited better prognoses compared with those in high-risk group. ROC curve analysis in training set and validating set showed that the sensitivity and specificity of the prognostic model were relatively favorable. Univariate and multivariate cox regression analyses confirmed the risk score computed by our prognostic model was an independent prognostic factor for HCC patients. Furthermore, GSEA between high-risk and low-risk group of HCC patients significantly enriched multiple oncological pathways, various biosynthesis and metabolic process, which might explain the biological functions and molecular mechanisms of the prognostic model based on SFs.

In the prognostic model constructed in our study, DNAJC6 and IGF2BP3 were risk factors, while ZC3H13 and DDX19B were protective factors. DNAJC6 (DNA/HSP40 homolog subfamily C member 6) encodes the brain‐specific isoform of auxilin. Auxilins is essential for the clathrin‐mediated endocytosis (CME), which is crucial for material uptake of cells through clathrin‐coated vesicles. Previous study has reported that two uncommon noncoding DNAJC6 variants may regulate RNA splicing, and DNAJC6 mutations is involved in autosomal recessive and early‐onset Parkinson's disease^32^. Another study observed DNAJC6 was significantly upregulated in HCC and significantly correlated with tumor progression and poor outcome of HCC patients. Mechanically, DNAJC6 facilitates transforming growth factor β (TGF-β) pathway activation to promote epithelial-mesenchymal transition (EMT), thereby promotes HCC cell proliferation and invasion^33^. IGF2BP3 is a member of the insulin-like growth factor 2 mRNA binding protein family. It has been confirmed that upregulation of IGF2BP3 promotes initiation and progression of multiple cancers, such as bladder cancer and colon cancer. In bladder cancer, IGF2BP3 was reported to enhance cell proliferation and inhibit cell apoptosis through activation of JAK/STAT pathway^34^. In colon cancer, IGF2BP3 binds to the mRNA of CCND1 and VEGFA via recognizing m6A modification of CCND1 and VEGFA, and enhances their mRNA stability, which facilitates cell proliferation and angiogenesis respectively^35^. A recent study has confirmed IGF2BP3 directly regulates alternative splicing of PKM and BTF3 and thus contributes to lung tumorigenesis^36^. ZC3H13 (zinc finger CCCH domain‐containing protein 13), a classical CCCH zinc finger protein, inhibits proliferation and invasion of colorectal cancer cells via blocking the Ras-ERK signaling pathway^37^. DDX19B (DEAD-box Helicase 19 B) participates in regulating mRNA export and mRNA translation^38^. To date, the role of DDX19B in cancers remains unclear. Collectively, the roles of DNAJC6, IGF2BP3, and ZC3H13 in regulating cancer progression as mentioned in above studies are consistent with our present study, indicating the results based on our study are reliable.

However, there is limited research on the roles of DNAJC6, IGF2BP3, ZC3H13, and DDX19B in the regulation of AS events. Therefore, we explored the correlations between these SFs (DNAJC6, IGF2BP3, ZC3H13, and DDX19B) and prognostic AS events. Then we extracted protein-coding genes from AS events regulated by SFs mentioned above for further functional exploration. seven pathways (glyoxylate and dicarboxylate metabolism, primary bile acid biosynthesis, complement and coagulation cascades, PPAR signaling pathway, tryptophan metabolism, propanoate metabolism, and prion disease) were enriched by both GSEA of our prognostic model and KEGG pathway analysis of protein-coding genes of AS events associated SFs in the prognostic model. It has been reported dysregulation of glyoxylate and dicarboxylate metabolism is involved in gastric cancer and colorectal cancer^39,40^. Complement and coagulation cascades has been confirmed to be associated with chemosensitivity and overall survival of patients with soft tissue sarcoma^41^. PPAR (peroxisome proliferator-activated receptor) is a canonical *pathway* involved in lipid metabolism. PPAR family, composed of three transcription factors (PPARα, PPARβ/δ, and PPARγ), controls energy and metabolism balance^42^. The anticancer effect of PPAR has been elucidated in multiple cancer, such as gastric cancer and lung cancer^43,44^. Tryptophan (TRP) is implicated in neuronal function, immunity, and gut homeostasis, etc. The imbalance in the synthesis of TRP metabolites has been demonstrated to be associated with neurologic and psychiatric disorders, chronic immune activation and immune escape of cancers^45^. Thus, we speculated ZC3H13, IGF2BP3, and DDX19B might participate in the occurrence and development of HCC through regulating their correlated AS events and inducing dysregulation of above cancer-related pathways.

There were several limitations in this study. Firstly, the prognostic model based on SFs was only verified in the internal data of TCGA but not verified in external independent cohorts. Secondly, the prognostic model based on SFs is not yet clinically validated. Thirdly, the regulatory relationship among SFs and AS events were established through statistical correlations, and further biological experiments are needed to verify the exact AS events regulated by ZC3H13, IGF2BP3, and DDX19B. Forth, the biological functions and molecular mechanisms of the prognostic model implicated in HCC progression are preliminary explored by bioinformatic analysis, which also need large amounts of biological experiments to validate in the future.

Taken together, we established an independent and robust prognostic model based on prognosis-associated SFs, providing novel targets for diagnosis, prognosis, and therapy of HCC. In addition, we constructed the prognostic SFs-AS events regulatory network, and explored the potential roles of SFs via modulating AS event in HCC, which paved the way for seeking novel biological functions and molecular mechanisms of SFs in HCC tumorigenesis and progression.

## Supplementary Information

Supplementary Table S1.

Supplementary Table S2.

Supplementary Table S3.

Supplementary Table S4.

Supplementary Table S5.

Supplementary Table S6.

Supplementary Table S7.

Supplementary Table S8.

## Data Availability

Gene expression data and clinical information of HCC can be accessed in TCGA. The alternative splicing events data of HCC can be accessed in TCGA SpliceSeq.

## Code availability

All code used during the study are available from the corresponding author by request.
